# Mathematically Derived Body Volume and Risk of Musculoskeletal Pain among Housewives in North India

**DOI:** 10.1371/journal.pone.0080133

**Published:** 2013-11-06

**Authors:** Vipin Bihari, Chandrasekharan Nair Kesavachandran, Neeraj Mathur, Balram Singh Pangtey, Ritul Kamal, Manoj Kumar Pathak, Anup Kumar Srivastava

**Affiliations:** Epidemiology Division, CSIR-Indian Institute of Toxicology Research, Lucknow, Uttar Pradesh, India; Universidad Pablo de Olavide, Centro Andaluz de Biología del Desarrollo-CSIC, Spain

## Abstract

**Background:**

Global Burden of Disease Study 2010 demonstrates the impact of musculoskeletal diseases as the second greatest cause of disability globally in all regions of the world. The study was conducted to determine the role of mathematically derived body volume (BV), body volume index (BVI), body mass index (BMI), body surface area (BSA) and body fat % (BF %) on musculoskeletal pain (MSP) among housewives in National Capital Region (NCR).

**Methods:**

A cross sectional study was undertaken among 495 housewives from Gurgaon and New Okhla Industrial Development Area (NOIDA) in National Capital Region (NCR), New Delhi, India. The study includes questionnaire survey, clinical examination and body composition monitoring among housewives.

**Results:**

A significantly higher BMI, BVI, BV and BSA were observed in subjects with MSP as compared to those who had no MSP. This was also true for subjects with pain in knee for BMI category for overweight. Subjects with pain in limbs had significantly high BMI and BVI as compared to subjects with no MSP. A significant positive correlation of age with BMI, BVI, BV and BSA was observed among subjects having no MSP denoting a direct relationship of age and these body factors.

**Conclusions:**

The prevalence of MSP among housewives is associated with increasing age, BMI and BVI. This can possibly be used for formulating a strategy for prevention of MSP.

## Introduction

Musculoskeletal conditions affect more than 1.7 billion people worldwide and has the 4^th^ greatest impact on the overall health of the world population, considering both death and disability. This burden has increased by 45% during the past 20 years and will continue to escalate unless action is taken [1]. These cause considerable functional limitations in the adult population in most welfare states than any other group of disorders. They are also a major cause of years lived with disability (YLDs) in all continents and economies [2].

Current estimates of people affected worldwide include: back pain 632 million, neck pain 332 million, osteoarthritis of the knee 251 million, and other musculoskeletal (MS) conditions 561 million. As a group MS disorders cause 21.3% of all YLDs, and is second only to mental/ behavioral disorders that account for 22.7% of YLDs. Worldwide low back pain is leading cause of disability and contributes 10.7% of total YLDs. Low back pain (83.1 million YLDs), neck pain (33.6 million YLDs), and osteoarthritis (17.1 million YLDs) are chief causes of MS problems [1].

The housewives form the core that nurtures the society. They perform a multitude of tasks that cause ergonomic stress as well as exhaustion of muscle groups that result in MSP. Deviations from the optimum body composition is likely to precipitate and exacerbate MSP from various causes. Scant information is available about the relationship of body composition factors like BV, BF % vis a vis MSP in the Indian context.

BMI is a commonly used indicator for screening of body composition. It is widely used to predict ideal weight in relation to height and identify malnourished individuals and groups. BMI, however, has many limitations. Firstly, it does not actually measure the percentage of body fat and hence, fails to precisely pin-point the weight distribution across the body. Secondly, as it is heavily dependent on height and weight variables, it leads to simplistic and often grossly incorrect assumptions regarding the distribution of muscles and bone mass. Thirdly, the BMI’s efficacy is poor in higher age groups due to the loss of height in old age. This implies that the BMI in such cases increases despite the absence of a corresponding increase in weight – a major fallacy [1].

BVI was designed and developed in 2000 as a computer based three dimensional scan of the human body with regards to obesity and as a potential substitute for the BMI, producing far superior and accurate results. Since then a study has shown that BVI is a better indicator as compared to BMI [1]. As of date we are not aware of any studies from Asian countries. The reason may lie in the fact that 3D body scanners are scantily available. Moreover, even studies of mathematically derived BV in relation to MSP are not available.

We earlier reported that 35.9 % of housewives in NCR suffered from MSP [3]. The Global Burden of Diseases study 2010 [4] confirmed our findings. Scanty information is available in existing literature regarding the role of body composition factors and occurrence of MSP. The study was performed to explore the role of mathematically derived BV, BVI and BF% in MSP among housewives in NCR.

## Methods

### Study sample and selection process

Four hundred and ninety five housewives from Gurgaon and NOIDA in National Capital Region (NCR), New Delhi, India were studied. Households were selected using systematic random sample using the list of households from governmental agencies in these areas as sampling frame. All the identified residents were approached through household visits by social workers and requested to participate in the study on the basis of voluntary informed consent. The participation of the study subjects was ensured by motivating the community leaders, teachers, doctors etc through social workers. Ninety per cent of the registered population turned up for the study. The volunteers were free to withdraw their participation from study at any stage.

### Inclusion criteria

1. Age group of housewives must be 18 - 90 years.
2. Women who are married and stay at home.
3. Not involved in any economically productive occupation.
4. Women doing housework like cooking, washing etc.

### Exclusion criteria

1. Female who are employed.
2. Age group below 18 years and above 90 years.
3. Pregnant women.
4. Those not meeting inclusion criteria 2, 3, 4.

### Ethical Statement

Ethical clearance was obtained from CSIR-Indian Institute of Toxicology Research- Institutional Human Ethical Committee (IHEC), Lucknow, India before starting the study. The committee follows Indian Council of Medical Research guidelines for biomedical research on human participants according to principles expressed in the Declaration of Helsinki. Written informed consent was taken from participants. It was recorded in local language on a consent form (also in local language) approved by CSIR-Indian Institute of Toxicology Research- Institutional Human Ethical Committee (IHEC), Lucknow, India. The ethics committee also approved the consent procedure and study-information brochure.

## Study Parameters

### Detailed History

Personal, social and occupational details of each subject were collected through structured interview method and all information was noted on a pretested questionnaire. The personal details comprised information regarding age, dietary practices, smoking, addictions, education and income. 

### Clinical Examination

 Information regarding location and degree of pain was collected through structured interview. A pre-tested questionnaire was developed to include the information. The investigation depended on self-reported symptoms along with their location. A pain diagram was prepared showing a sketch of the human body in a standing posture (rear view) along with body locations marked with arrows. During the interview each subject was asked to pinpoint the body location(s) where pain was felt, at the time of interview or preceding 24 hours. Information about pain in the neck, shoulders, upper back, upper arms, lower back, forearms, wrists, hip/ buttocks, thighs, knees, lower legs and ankles was recorded for each individual. 

### Body composition measurements

 BMI and BF% was measured using bioelectric impedance analysis (BIA) based Body fat monitor (Model: Omron HBF-352, Omron Co. Ltd., Japan). Height was measured using Stadiometer and weight measured using Manual Personal Scale. 

The BMI of the subjects was classified into normal, overweight and obese based on WHO classification. BF% were graded for low, normal and high based on the earlier report [5]. 

BSA was calculated using DuBois and DuBois formula [6], 

BSA (m^2^) = 0.007184 x power [Height (cm), 0.725] x power [Weight (kg), 0.425] 

Body Volume Index -BVI (V/S) was calculated using Sendroy et al formula for female [7] and BV was calculated as product of BVI and BSA [7].

### Data Processing and Analysis

 Data recorded on a predesigned performa was entered in Microsoft Excel spreadsheet. All the entries were double checked for possible keyboard errors. Mean values of the body composition factors between different groups was compared using Student’s t-test. Pearson correlation was used to assess the association of age and BF% with the body composition factors (BMI, BVI, BV, BSA). Subjects with MSP (pain in upper back, pain in lower limb, pain in limbs, pain in lower back and pain in knee) among study subjects were compared for body composition factors with no MSP cases. Linear regression analysis was performed taking BVI as dependant variable and other body composition factors as the independent variables. The criterion of significance was set at *p*<0.05. All statistical calculations have been done using IBM SPSS Statistics ver. 20.

Among the 495 subjects studied, data of 387 subjects for BF % was available and used to calculate the attributable risk of MSP. The study subjects with normal BMI (as per WHO standard) and BF % (as per Omron Healthcare) were used to determine the mean values for BV and weight. These mean values were then used as cut off points for defining the control and for the calculation of attributable risk of MSP. The subjects having mean body volume and weight less than the mean value obtained from the group with normal BMI and BF% were considered to be control subjects. Attributable risk was calculated as difference between prevalence of MSP in cases and control subjects, using the formula AR%= [(Prevalence in cases – prevalence in control)/prevalence in control] x 100.

## Results

Routine activities of housewives and the possible physical stress are shown in Table 1. Physical characteristics, body composition and dietary habits of study subjects are presented in Table 2. The mean age, height and weight of the study subjects was 34.9 yr, 154.5cm and 52.1 kg respectively. BSA of study subjects were in lower limits from the proposed normal range (1.6 - 1.9 m^2^) [8]. 

**Table 1:** Type of activity among housewives and their possible physical stress.

**Type of activity**	**Physical stress**
Cooking (biomass fuel based stove)	Bending and stretching of arm and leg muscles, since stoves are usually placed on the ground.
Cooking (electric/ gas stove)	Stretching and bending of arms for cooking, since stoves are generally placed on platforms.
Cleaning of utensils/clothes	Involvement of hands and arm muscles for washing utensils/clothes.
Cleaning of rooms	Involvement of hands and arm muscles together with whole body muscular movement.
Parental care	Care of infant including holding the baby on the shoulder etc requires a lot of muscular movement.

**Table 2:** Physical characteristics, diet and body composition in study subjects (n = 495).

**Parameter**	**Mean ± SD**
Age (yrs.)	34.9±15.0
Height (cm.)	154.5±6.7
Weight (kg)	52.1±12.0
Body Mass Index (kg/cm^2^)	21.8±4.8
Body Surface Area (m^2^)	1.5±0.2
Body Volume (L)	50.0±11.7
Body Volume Index	33.3±4.1

More than 80% of the subjects were vegetarian. 37.4% of study subjects are illiterate. Details are shown in Table 3. However dietary habits and literacy was not found to be significantly associated with MSP.

**Table 3:** Socio-economic status of study subjects (n = 495).

**Variables**	**Sub category**	**Result n (%)**
Educational qualification	Illiterate	185 (37.4)
	Primary	66 (13.3)
	Junior High School	45 (9.1)
	High School	101 (20.4)
	Intermediate	54 (10.9)
	Graduate	28 (5.7)
	Post graduate	16 (3.2)
Diet	Vegetarian	403 (81.4)
	Non-vegetarian	92 (18.6)

Thirty one percent of housewives suffered from MSP. The percentage of subjects with pain in the body is shown in Table 4. Pain in lower back (10.7%) and upper back (7.9%) were commonly encountered among them. A significantly higher BMI, BVI, BV and BSA were observed in subjects with MSP as compared to those who had no MSP (Table 5). This was also true for subjects with pain in knee for BMI (over weight category as per WHO norms), also BVI, BV and BSA were found to be significantly higher as compared to subjects with no MSP. Subjects with pain limbs had significantly high BMI and BVI as compared to subjects with no MSP (*p*<0.05). Pain in back (both upper back and lower back) was not found to be affected by the body composition factors. 

**Table 4:** Morbidity profile of Musculoskeletal Pain (n = 495).

**Musculoskeletal pain**	**n (%)**
No MSP	341 (68.9)
Overall MSP	154 (31.1)
Pain in upper back	39 (7.9)
Pain in limbs	36 (7.3)
Pain in knee	20 (4.0)
Pain in lower back	53 (10.7)

MSP: Musculoskeletal Pain

**Table 5:** Musculoskeletal problem and its relation to body composition factors.

**Musculoskeletal problem**	**BMI** Mean ± SD	**BVI** Mean ± SD	**BV** Mean ± SD	**BSA** Mean ± SD
No MSP (n=341)	21.3±4.8	32.9±4.2	49.0±11.8	1.5±0.2
Overall MSP (n=154)	22.9±4.6 **	34.2±3.9 **	52.1±11.1 **	1.5±0.2 *
Pain in lower back (n=53)	21.8±3.9	33.4±3.4	52.3±9.4	1.5±0.1
Pain in knee (n=20)	25.9±5.1 **	36.9±4.1 **	59.4±11.7 **	1.6±0.2 *
Pain in limbs (n=36)	23.3±3.5 *	34.4±3.0 *	51.2±8.4	1.5±0.1
Pain in upper back (n=39)	22.7±5.1	33.9±4.1	51.7±12.2	1.5±0.2

* p<0.05, ** p<0.01, MSP: Musculoskeletal Pain

BMI: Body Mass Index, BVI: Body Volume Index, BV: Body Volume, BSA: Body Surface Area.

**Figure 1:**
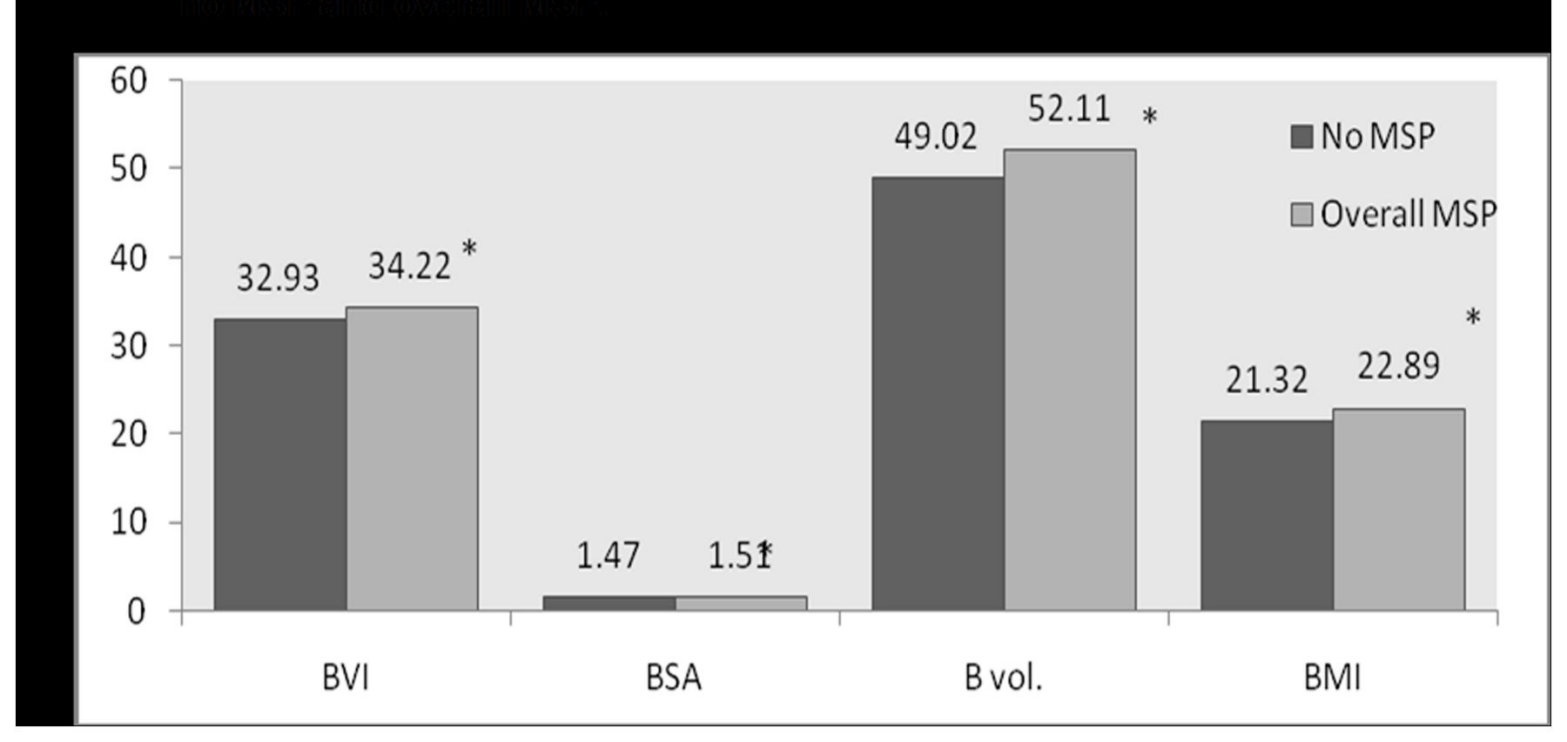
Bar graph for comparison of mean values of body composition factors between no MSP and overall MSP.

**Figure 2:**
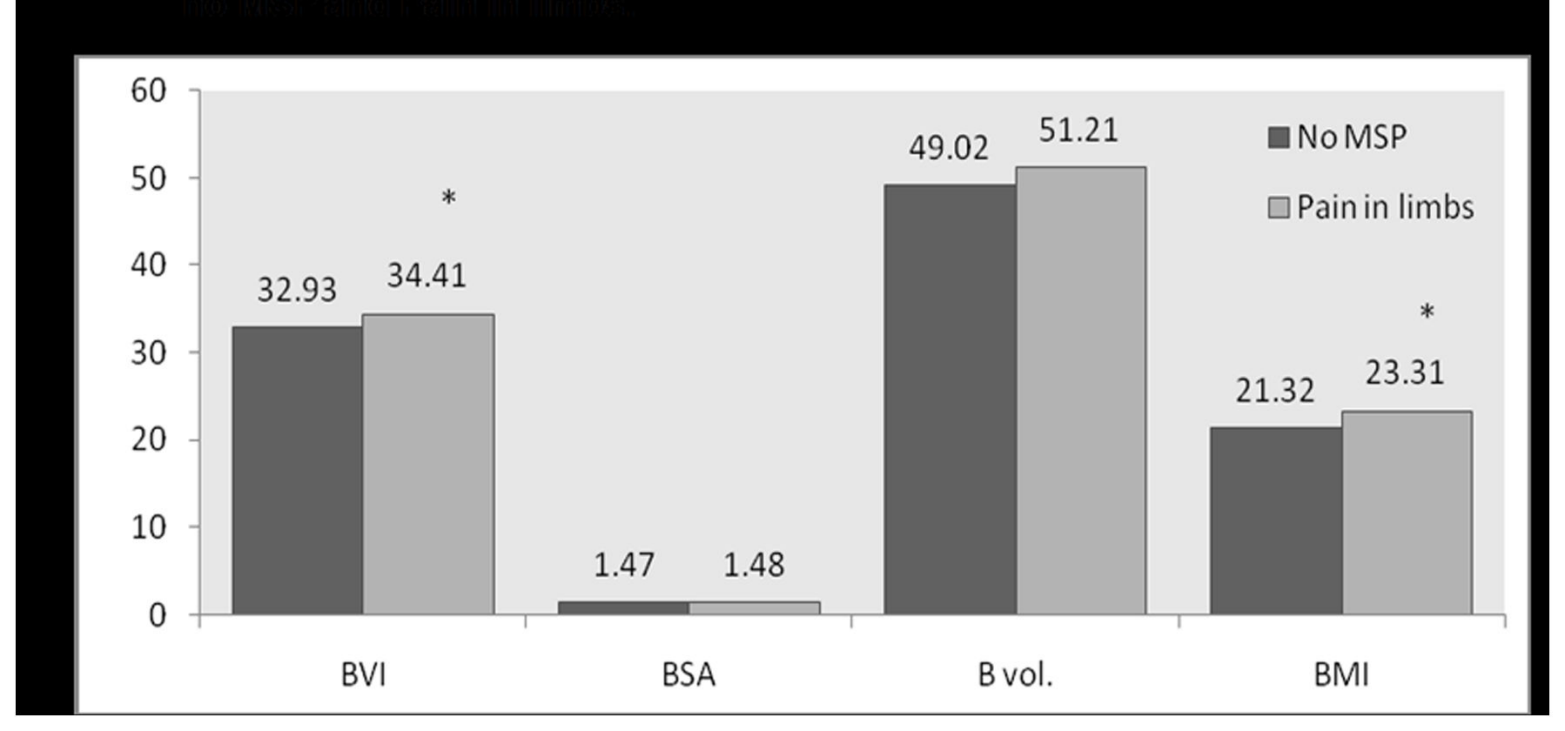
Bar graph for comparison of mean values of body composition factors between no MSP and Pain in limbs.

**Figure 3:**
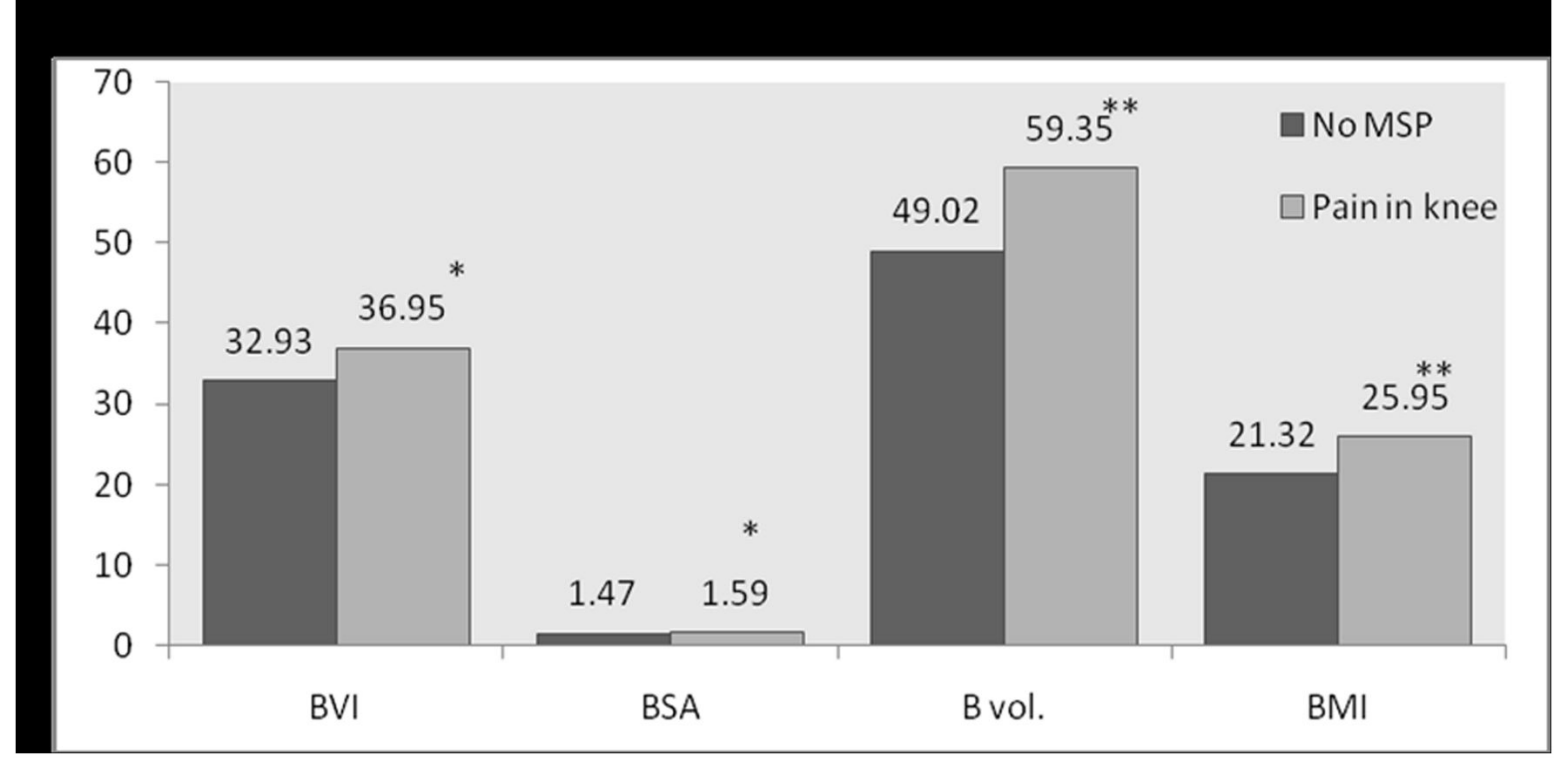
Bar graph for comparison of mean values of body composition factors between no MSP and Pain in knee.

Figure 1 shows significant increase in mean values of all the body composition factors (*p*<0.05) with respect to subjects with MSP complaints and no MSP. A significant increase in BVI and BMI (*p*<0.05) of subjects with pain in limbs compared to those with no MSP was detected (Figure 2). A significant increase in BVI, BSA, BV, and BMI of subjects with pain in knee as compared to those with no MSP (Figure 3) was observed. No statistically significant differences could be found in the mean values of body composition factors among subjects with pain in upper back and/or lower back as compared to subjects with no MSP.

**Figure 4:**
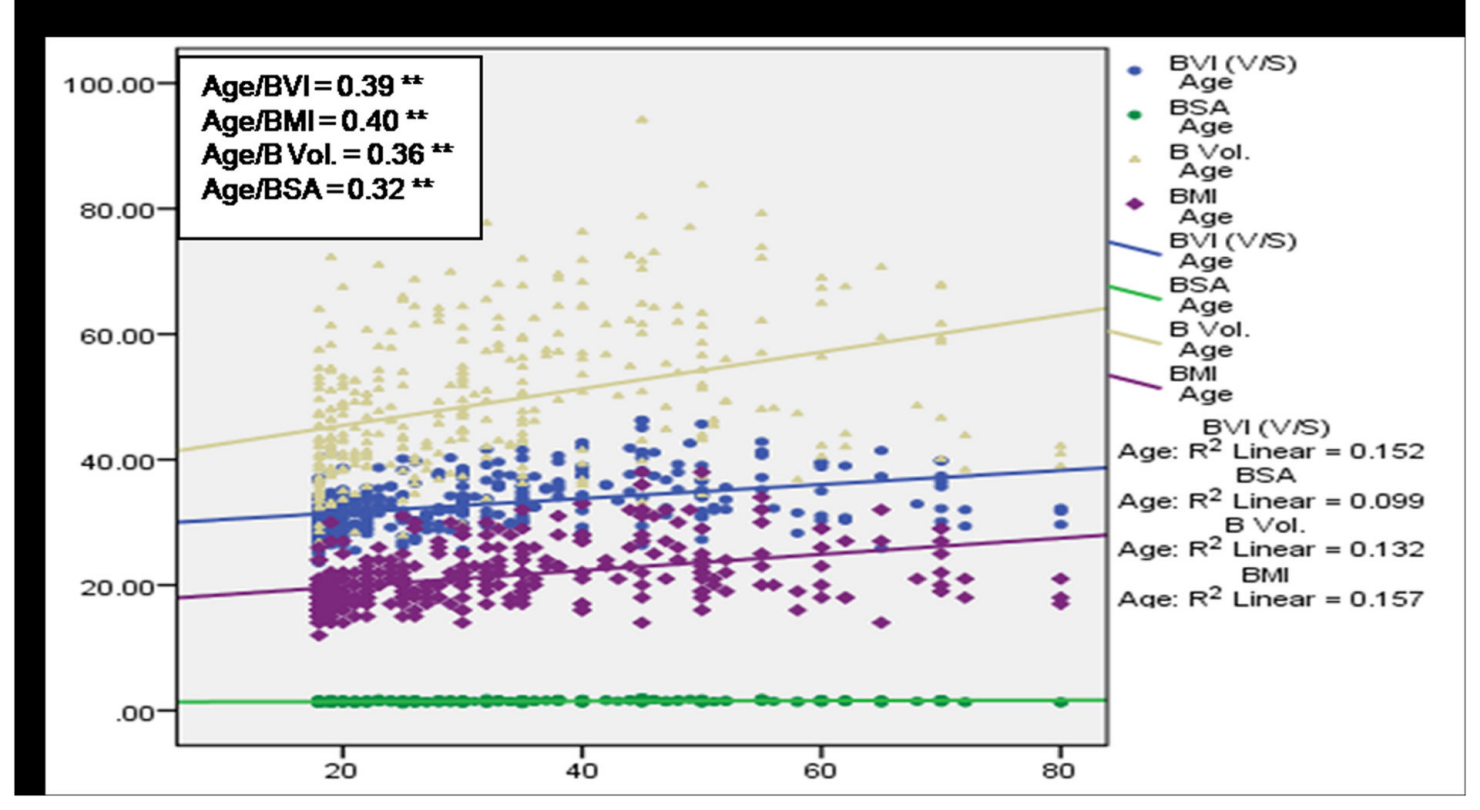
Scatter plot with correlation coefficients of age with various body composition factors in respect to no MSP (n = 341).

**Figure 5:**
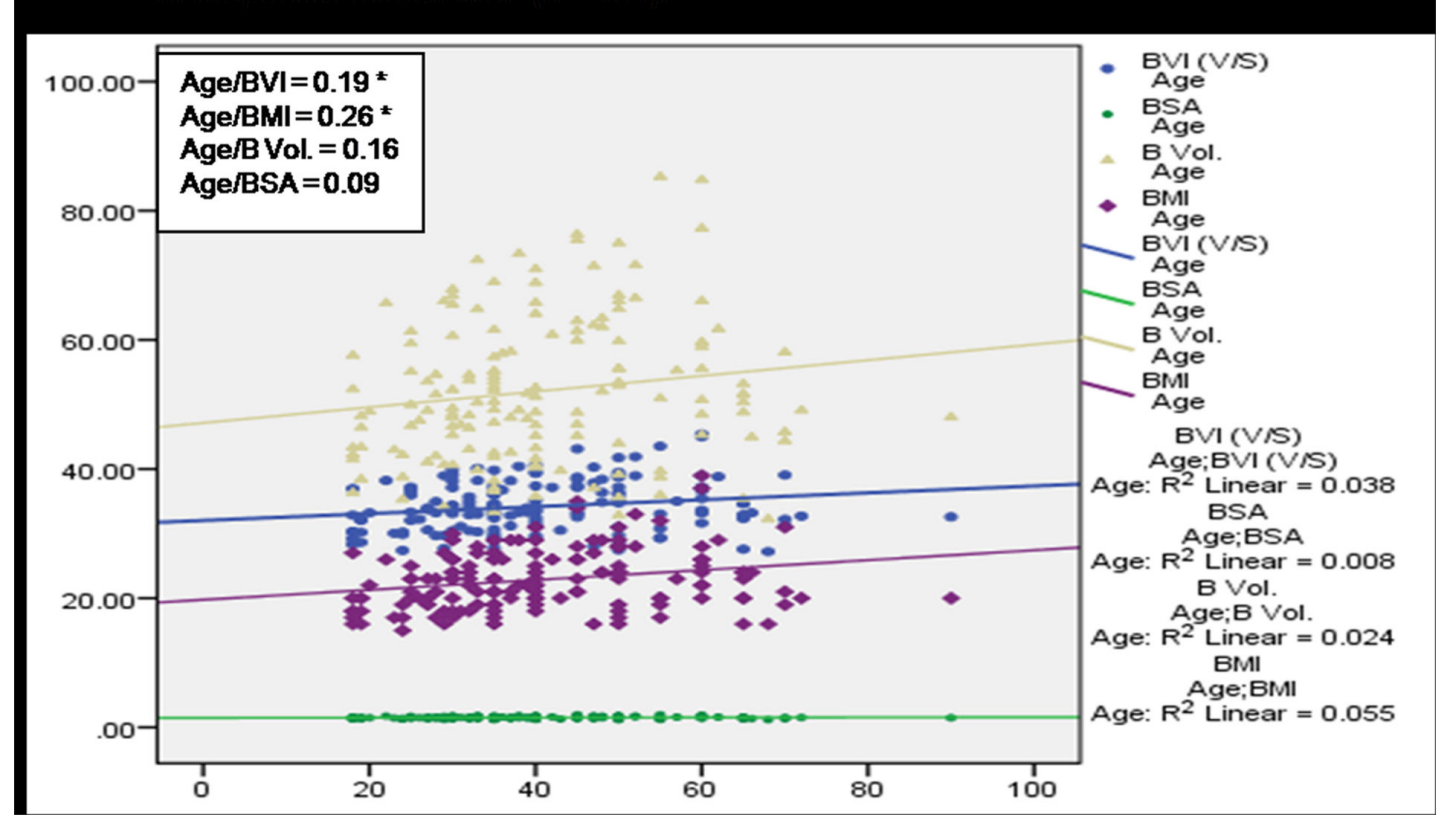
Scatter plot with correlation coefficients of age with various body composition factors in respect to overall MSP (n = 154).

**Figure 6:**
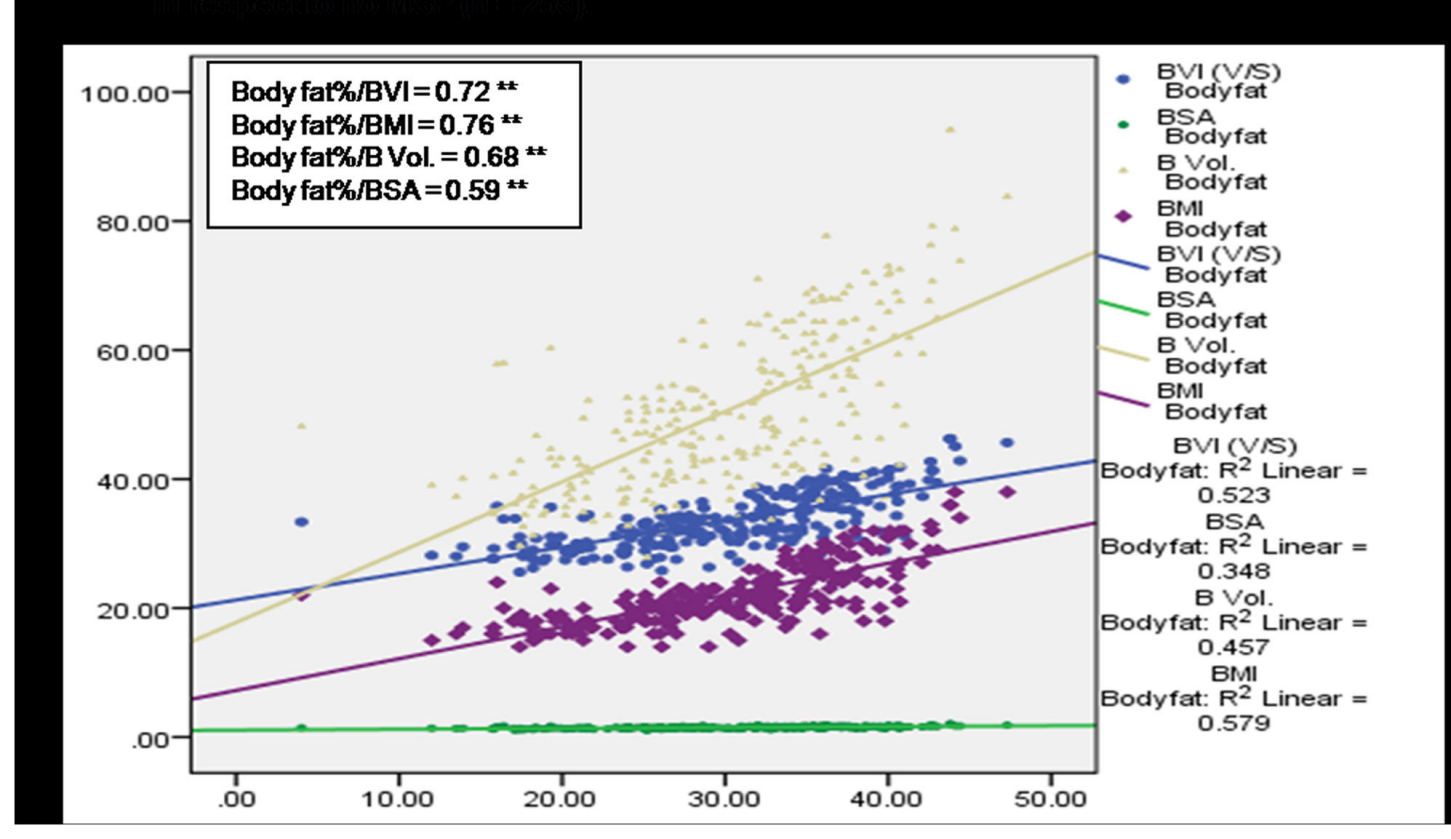
Scatter plot with correlation coefficients of body fat% with various body composition factors in respect to no MSP (n = 259).

**Figure 7:**
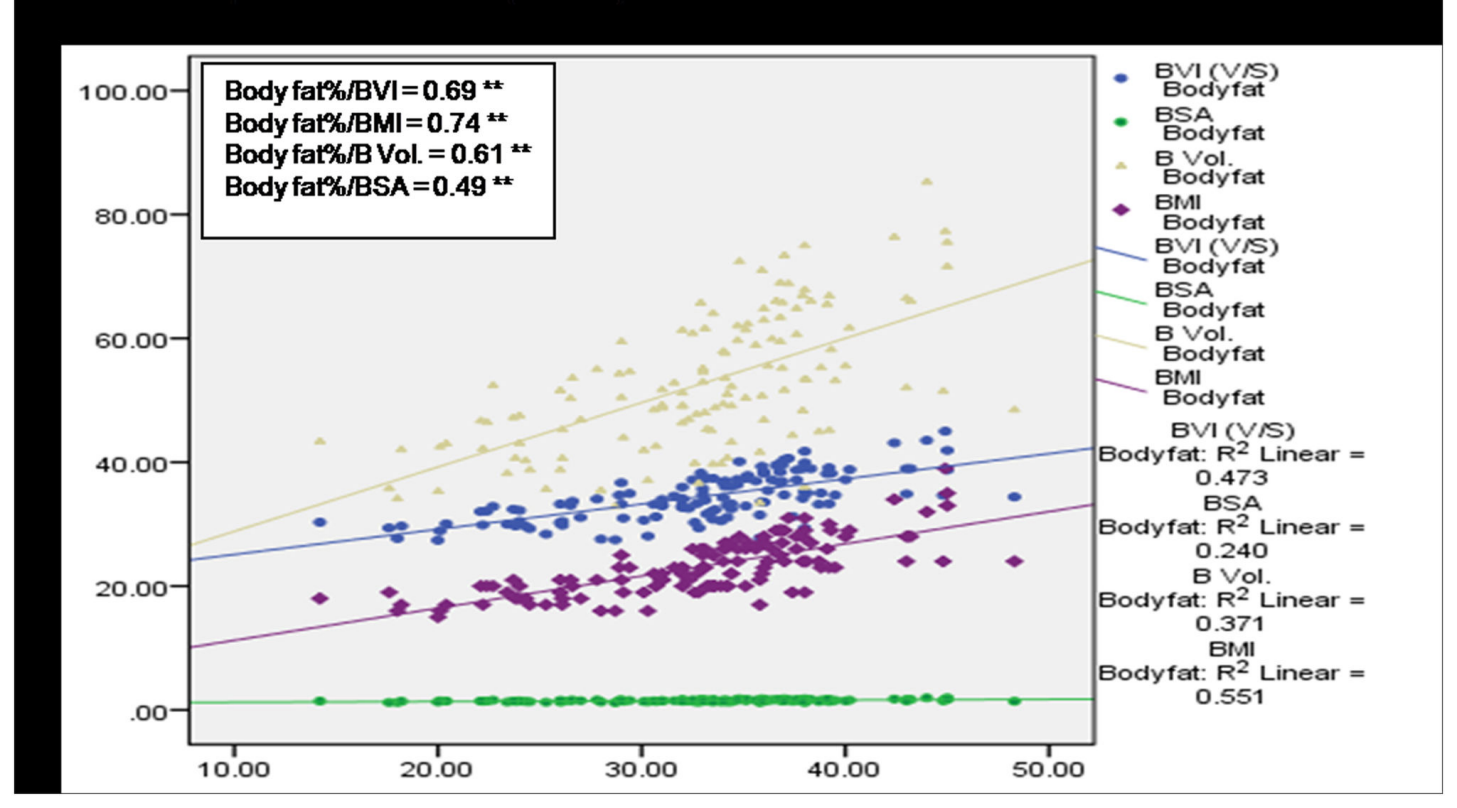
Scatter plot with correlation coefficients of body fat% with various body composition factors in respect to overall MSP (n = 128).

A significant (*p*<0.01) positive correlation of age with BMI, BVI, BV and BSA was observed among subjects having no MSP (Figure 4) denoting a direct relationship of age and these body factors. BVI, BMI and BV were found to increase with age among cases with MSP, however a statistically significant (p<0.05) association was observed only in case of BVI and BMI (Figure 5). Figure 6 and 7 depict highly significant positive correlation of BF % with all the body composition factors (*p*<0.01) among subjects having no MSP and a highly significant (*p*<0.01) positive correlation of body fat% with all the body composition factors among study subjects with MSP respectively. This shows that increase in body composition factors (BMI, BVI, BSA, BV) is directly proportional to BF % among study subjects.

**Figure 8:**
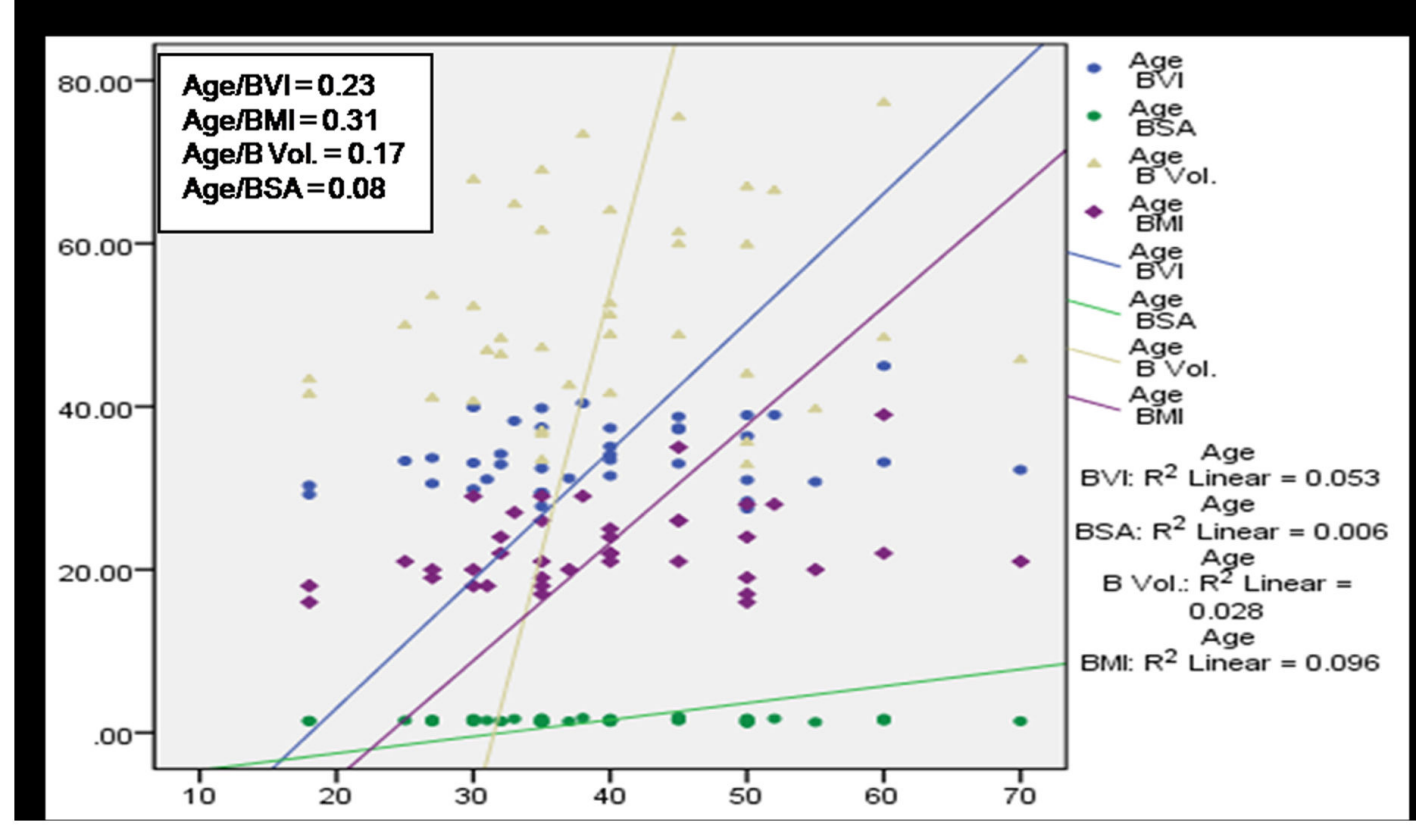
Scatter plot with correlation coefficients of age with various body composition factors in respect to pain in upper back.

**Figure 9:**
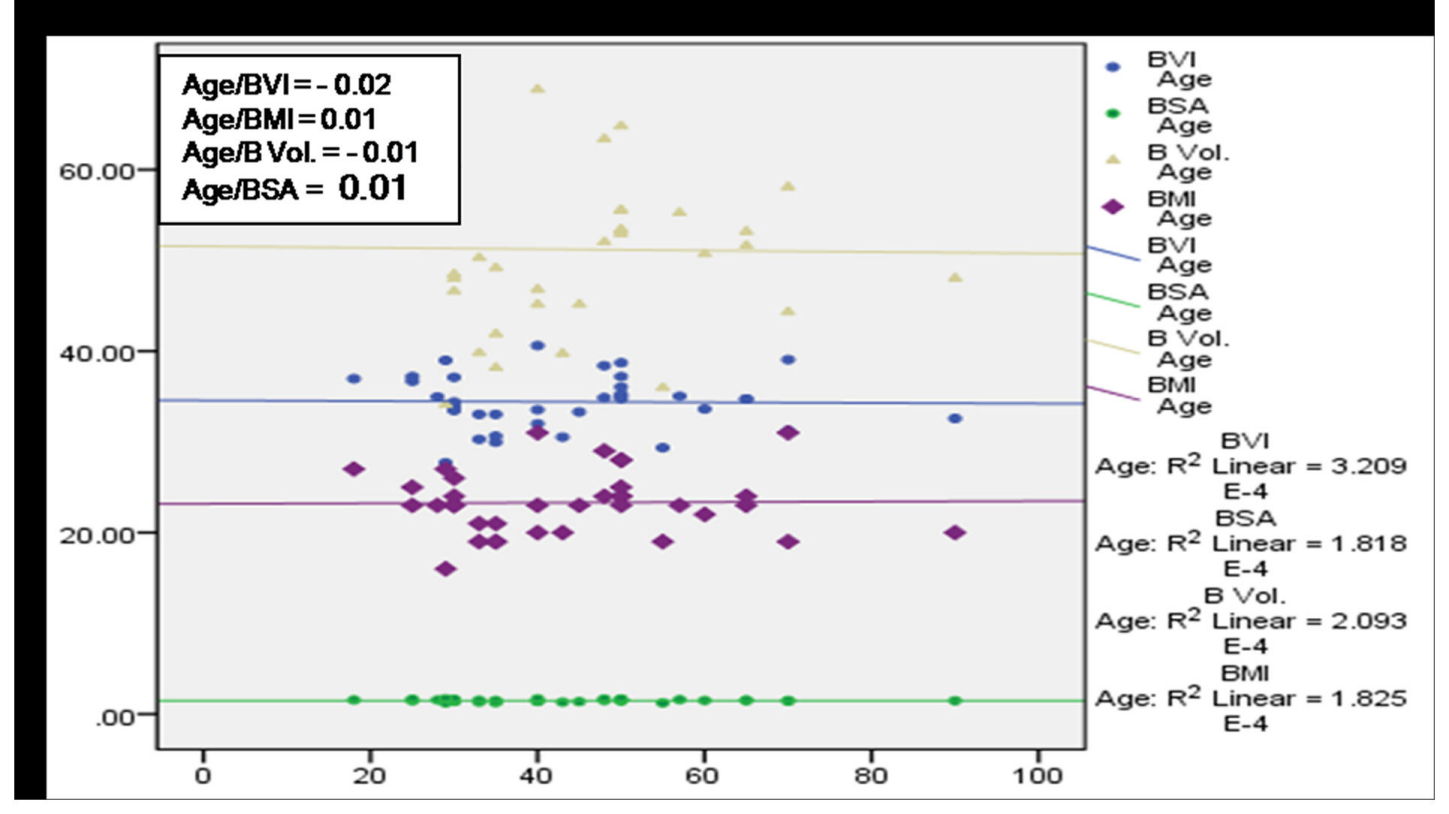
Scatter plot with correlation coefficients of age with various body composition factors in respect to pain in limbs.

**Figure 10:**
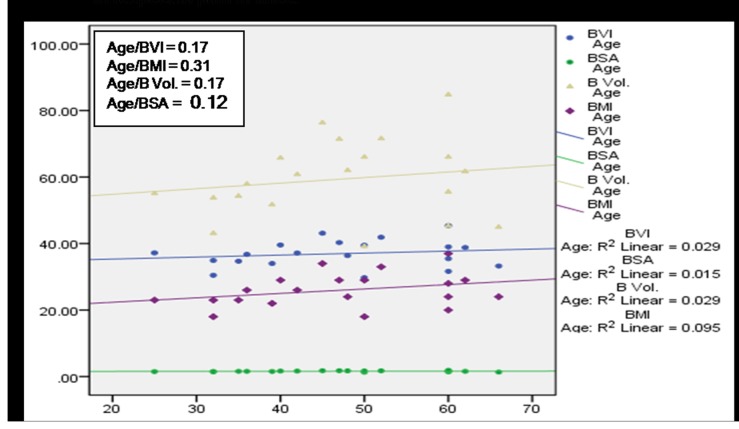
Scatter plot with correlation coefficients of age with various body composition factors in respect to pain in knee.

**Figure 11:**
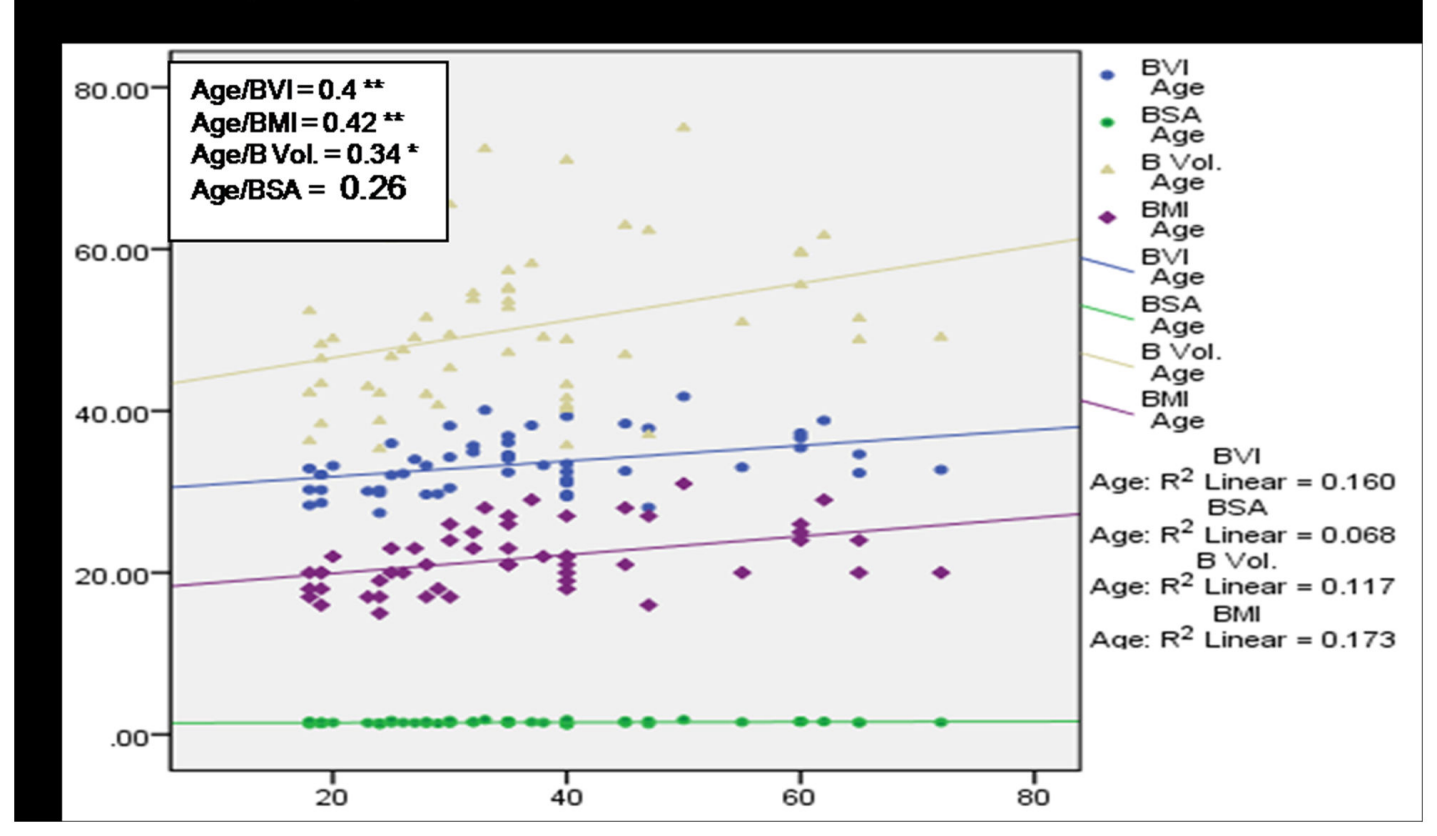
Scatter plot with correlation coefficients of age with various body composition factors in respect to pain in lower back.

A positive correlation between age and body composition factors in subjects with pain in upper back was observed (Figure 8). Figure 9 shows negative correlation of age with BVI and BV and positive correlation of age with BMI and BSA among subjects with pain in limbs. Figure 10 shows positive correlation between age and body composition factors with respect to subjects with pain knee. Figure 11 shows significant positive correlation of age with BVI, BMI and BV in subjects with pain in lower back. 

It was also calculated that 35.71% of the MSP can be attributed to increase in BV and 40.74% of the MSP can be attributed to increase in body weight of the subjects (shown in Tables 6 and 7). Based on the results of present study, if the BV and weight of housewives in NCR is in the range of cutoff points used for the calculations i.e. weight 50.6 kg and volume 48.6 L, then there would be a 40.74% and 35.71% reduction in the MSP respectively. Table 8 shows the linear regression analysis among the body composition factors taking BVI as the dependant variable. Significant positive correlation was observed between BVI and other body composition factors. Coefficient of determination (R^2^) was in the range of 81% to 95% for the analysis.

**Table 6:** Attributable risk of Musculoskeletal Pain for BV.

**Subjects**	**MSP n**	**No MSP n**	**Total n**	**Prevalence of MSP**	**Attributable risk% **
Control (BV ≤48.57)	50	130	180	P_u_ = 0.28	35.71
Cases (BV >48.57)	78	129	207	P_e_ = 0.38	
Total	128	259	387		

P_u_: Prevalence in controlP_e_: Prevalence in casesMSP: Musculoskeletal Pain, BV: Body Volume

**Table 7:** Attributable risk of Musculoskeletal pain (MSP) for weight (in kg).

**Subjects**	**MSP n**	**No MSP n**	**Total n**	**Prevalence of MSP**	**Attributable risk %**
Control (Weight ≤ 50.61)	47	128	175	P_u_ = 0.27	40.74
Cases (Weight > 50.61)	81	131	212	P_e_ = 0.38	
Total	128	259	387		

P_u_: Prevalence in controlP_e_: Prevalence in casesMSP: Musculoskeletal Pain

**Table 8:** Linear regression of BVI with various body composition factors.

**Parameter**	**Constant**	**β coefficient**	**r value**	**R^2^**
BV	15.98	0.35	0.98	0.95
BMI	15.04	0.84	0.96	0.93
BSA	0.004	22.5	0.90	0.81

BVI: Body Volume Index, BMI: Body Mass Index, BV: Body Volume, BSA: Body Surface Area

## Discussion

The present study confirms the fact that prevalence of MSP among housewives is associated with increasing age, BMI and BVI. Housewives perform diverse functions that are summarily mentioned in Table 1. These are the cause of increased ergonomic stress. Other physiological and medical causes also contribute in the causation of MSP. Earlier studies have been conducted to investigate the relationship between MSP and occupation like textile industry workers [9] and teachers [10]. However, no studies are available on these issues among housewives. Obesity has been associated with increased prevalence of low back pain in a systematic review [11]. A few prospective cohort studies have shown that exercise may reduce the risk of musculoskeletal pain [12-14], while other studies have found moderate or no associations [15].

A recent study showed that overweight and obesity increased the risk of widespread chronic musculoskeletal pain during 11-year follow-up, whereas physical exercise could compensate for this adverse effect to some extent [16]. Whether physical exercise and excess body mass have a similar effect on risk of localized chronic pain in the low back or neck/shoulders is unknown as per another study [17]. In the area of pain specifically, researchers have begun to examine whether increased weight may be associated with conditions like headache [18], fibromyalgia [19] and rheumatoid arthritis [20]. However the problem of overweight/ obesity and its association with pain is a question that has not been satisfactorily answered; no studies have been published to date in Indian context using BVI and BV. Use of three dimensional scanners will give more insights into the body composition and its relation to MSP.

Menopause is reported to be associated with increase in fat mass, decrease in lean mass and reduced output of ovarian steroids [21,22]. Several studies have reported that the higher prevalence of low back pain in menopausal middle aged women [23-25]. 

Increase in BMI, BF %, BV, BSA and BVI was observed among housewives with MSP as compared to no MSP group. No reports are available to compare our findings. The etiology of MSP is now generally accepted to be multi-factorial, encompassing physical and social influences. Age, sex and working in wrong posture, lifting of loads and other physically strenuous work increases the risk of MSP [26].

Different anthropometric measurements have different accuracy in estimating body fat distribution [27-29] and total body fat mass [27,30]. Measurements of abdominal fat layers most often measure abdominal, visceral and subcutaneous fat compartments. These measurements need multi-compartment methods for better evaluation. Measurements using BIA are quick, reliable and easy to compute [27,31]. BMI is thought to have moderate accuracy in measuring total body fat mass, but has difficulties in predicting body composition or body fat distribution [27,30]. Three dimensional computerized scanning based BV and BVI values are the gold standard. Alternatively, mathematically derived BV and BVI values may be used.

The problem of MSP is responsible for much misery and disability. Further it is often a reason for misuse of analgesics and anti-inflammatory drugs resulting in conditions ranging from stomach upset to renal failure and contributes to YLDs. Higher prevalence of MSP was observed with increase in age, along with other health problems in housewives. Similar MSP problems were observed among housewives in Bangladesh [32] and Kuwait [33]. The incidence of MSP was highest in housewives in age group 35-44 yrs compared to weavers and cultivators from Bangladesh [32]. The most common cause of MSP was non-specific low back pain, followed by fibromyalgia and knee osteoarthritis [32]. Musculoskeletal conditions are prevalent and their impact is pervasive. They are the most common cause of severe long term pain and physical disability, and they affect hundreds of millions of people around the world. They significantly affect the psychosocial status of people affected as well as their families and careers [2].

Musculoskeletal pain is also one of the most common reasons for seeking medical advice in Western societies [34,35]. Pain in the neck/shoulders and pain in the low back constitute the majority of all musculoskeletal disorders [36]. The economic cost of low back pain alone in Netherlands has been estimated as 1.7% of the gross national product [37]. Nearly 60 per cent of the people in India have significant back pain at some time or the other during their lives [38]. Knowledge of the incidence of musculoskeletal disorders and their burden is fundamental to any country’s health-planning [39]. 

As age advances, women tend to gain weight and an additional burden is placed on the lower back causing various types of disabilities including low back pain [40]. While it is difficult to devise a preventive strategy for such a condition, our results in this population show that house wives having a mean BMI of 21.3 kg/cm^2^, BVI of 32.9, BV 49.0 L and BSA 1.5 m^2^ have the best chance of being pain free. In view of the present findings, more studies on the musculoskeletal pain among housewives from other parts of the country and further correlations with body composition factors are needed. With more such studies using objective measures like 3D body scans, safe ranges of BVI and BMI can be calculated for different ethnic groups. Awareness based on the present findings and combined with suitable fitness regimes can form the basis of a strategy for reducing the burden of MSP globally.
